# Hypersensitivity to Carboplatin in Children with Malignancy

**DOI:** 10.3389/fphar.2017.00201

**Published:** 2017-04-12

**Authors:** Antonio Ruggiero, Daniela Rizzo, Martina Catalano, Giorgio Attinà, Riccardo Riccardi

**Affiliations:** Division of Pediatric Oncology, Catholic University of RomeRome, Italy

**Keywords:** hypersensitivity reactions, carboplatin, children, management, desensitization

## Abstract

**Purpose:** Carboplatin-based regimens have proven efficacy in children with cancer. However, the development of hypersensitivity reactions (HSRs) may have a negative impact on treatment intensity and patients’ outcome. The aim of this review is to summarize the incidence and the clinical features of HSRs occurring in children with cancer treated with carboplatin and their impact on treatment efficacy.

**Methods:** Data were collected by searching for relevant studies on the incidence, clinical features and management of possible side effects about the use of carboplatin in children, published from March 1987 to October 2016 in the PubMed database.

**Results:** Carboplatin HSRs present with mild/moderate to severe clinical patterns. The risk of HSR is related to the cumulative number of infusions. Moreover, a greater risk of developing an HSR has been observed in younger patients than in older age groups of children; risk is also greater in girls and in patients with a prior history of allergy to other drugs. Management options include cessation of carboplatin and switching to another agent, premedication with antihistamines and/or corticosteroids, and carboplatin desensitization. For sensitized patients who have obtained benefits from carboplatin, the continuation of the treatment is desirable and desensitization protocols have showed promising results.

**Conclusion:** Clinicians must not underestimate the potential risk and occurrence of carboplatin HSRs in the pediatric population in order to outline adequate management strategies. Desensitization protocols should be considered for patients sensitive to carboplatin in order to avoid having to discontinue an effective chemotherapy.

## Introduction

Carboplatin is a second-generation platinum compound developed to reduce the side effects of cisplatin, particularly neurotoxicity, nephrotoxicity and emesis, while maintaining comparable antitumor activity and effectiveness. Carboplatin is widely used to treat solid tumors in adults, especially for ovarian and lung cancer, and for several types of malignancies in children such as brain tumor, neuroblastoma, retinoblastoma, germ cell tumors, and hepatoblastoma. Moreover, the treatment protocol based upon the combination of carboplatin and vincristine reported by Packer et al. (1997) seems to produce consistent and long-lasting responses in children with low grade glioma (LGG) (Chiaretti et al., 2004; Trisciuzzi et al., 2004). This is the most widely adopted chemotherapy for childhood LGG, offering high objective response rates in relapsed and newly diagnosed patients of 52 and 62%, respectively.

Carboplatin has been used with increasing frequency for the management of childhood cancers, and hypersensitivity reactions (HSRs) have consequently emerged as a significant complication of the therapy. Multiple exposures to this chemotherapeutic agent can cause sometimes life-threatening events, requiring discontinuation of treatment. However, while carboplatin-associated HSRs have been described extensively and analyzed in large cohorts of adult patients, our knowledge of their features in pediatric patients remains limited.

## Incidence

Allergic hypersensitivity to carboplatin is frequently reported in children, and the extensive use of carboplatin-based chemotherapy has brought with it an increase in allergic reactions (Allen et al., 1987; Chang et al., 1995; Lazzareschi et al., 2002). Carboplatin HSR has been described mostly in pediatric series of LGG, where the reported incidence is up to 47% depending on the schedules of administration (Lafay-Cousin et al., 2008; Dodgshun et al., 2016).

In the initial reports on the carboplatin-vincristine combination in pediatric LGG, Packer et al. (1997) observed a frequency of 7%. In a cohort of 29 children with LGG, Lazzareschi et al. (2002) reported six patients (20%) who developed HSRs to carboplatin. In the retrospective, cooperative Canadian study 42% of children with LGG who received a carboplatin-based chemotherapy regimen developed HSRs during the course of the treatment (Lafay-Cousin et al., 2008). Genc et al. (2012) observed a frequency of 40% in their study. Recently, carboplatin hypersensitivity was documented in 47% of patients by Dodgshun et al. (2016), and in the study by Shah et al. (2016) of 144 children with LGG treated with carboplatin and vincristine, 56 (39%) experienced an HSR to carboplatin.

## Clinical Features

Carboplatin can induce mild to moderate and severe HSRs which may develop acutely during infusion or within minutes, hours, or days after the drug has been delivered.

Clinical evidence of HSRs are graded 0 through 5 (grade 5 being death) by using the NCI Common Terminology Criteria for Adverse Events [CTCAE] (2010, CTCAE v. 4.03).

Mild and moderate reactions include all cutaneous reactions (flushing, pruritus, urticaria, angioedema, and maculopapular rash) not associated with symptoms affecting other organ systems. Severe reactions include chest pain, dyspnea, oxygen desaturation, edema/angioedema, changes in blood pressure and cardiovascular collapse.

Urticaria and facial rash may be the first and most common manifestations of hypersensitivity (Lazzareschi et al., 2002) and Markman et al. (1999) reported more than 50% of patients developing at least moderately severe symptoms. Symptoms usually resolved quickly with antihistamines and steroids.

More severe reactions and systemic anaphylaxis may be life-threatening (Cefalo et al., 2010). It is possible that carboplatin HSRs are not always recognized. Often, the early signs are subtle and may include only a mild rash and a mild bronchospasm (Wiesner et al., 2004). Patients should be alerted and appropriately instructed so that symptoms are promptly recognized and the diagnosis of HSR established to prevent potentially dangerous retreatment.

## Pathophysiology

The exact mechanism of carboplatin HSRs remains unclear, but the different clinical patterns of allergic reactions suggest that various immunological and non-immunological mechanisms are involved (Zanotti et al., 2001). Likelihood of type I IgE-mediated hypersensitivity increases with the rising incidence of hypersensitivity with repeated doses and with positive skin prick test reactions to platinum compounds (Leguy-Seguin et al., 2007). Carboplatin can act as a hapten and cause a type I IgE-mediated, histaminergic reaction with release of inflammatory molecules (Navo et al., 2006; Makrilia et al., 2010). Type I IgE-mediated hypersensitivity may be linked to early onset manifestations, such as itching, chest pain, rash, and anaphylactic reactions. An alternative to the type I IgE-mediated mechanism is type IV hypersensitivity, mediated by T cells, a delayed inflammatory reaction occurring hours or days after the infusion.

Some authors have tried to predict HSRs by using skin testing (Zanotti et al., 2001; Cefalo et al., 2010). Zanotti et al. (2001) developed a skin-test protocol for adult patients with gynecological malignancies and first demonstrated that skin testing for carboplatin made it possible to identify patients at risk for HSRs with a 99% negative predictive value. A cohort of 47 patients received a 0.02 ml intradermal injection of an undiluted aliquot of carboplatin 1 h before each course of chemotherapy. A negative skin test accurately predicted the absence of HSRs in 166 out of 168 courses of chemotherapy. Markman et al. (2003) performed skin tests on 126 women with gynecological cancer 30 min before each carboplatin treatment after the sixth cycle. They reported that skin tests had been positive in six out of seven patients who later developed anaphylaxis during carboplatin re-administration, finding a 98.5% negative predictive value. Therefore, a negative carboplatin skin test seems to predict with reasonable reliability the absence of a severe HSR with subsequent infusion of the drug. However, the implications of a positive test remain less certain and the limited experience with continued treatment suggests that this approach must be undertaken with considerable caution. According to Lazzareschi et al. (2002), the lack of sensitivity in skin tests could be explained by the fact that the molecular weight of carboplatin (373.272 g/mol) is low and it is not immunogenic in the native form.

## Risk Factors

A greater risk of developing an HSR has been observed in younger patients than in older age groups of children (Genc et al., 2012); risk is also greater in girls (Lafay-Cousin et al., 2008) and in patients with a prior history of allergy to other drugs (Ruggiero et al., 2010). No significant correlation was found between the occurrence of carboplatin HSRs and previous surgery, radiotherapy or tumor location (Genc et al., 2012).

The risk of hypersensitivity is related to the cumulative number of infusions rather than to the cumulative dose of carboplatin (Chang et al., 1995), thus it increases with repeated exposure to carboplatin (Schiavetti et al., 1999; Yu et al., 2001; Lazzareschi et al., 2002; Wiesner et al., 2004). The first HSR generally occurs between the 7th and the 10th carboplatin infusion (Schiavetti et al., 1999; Lazzareschi et al., 2002; Wiesner et al., 2004). Indeed HSRs are uncommon during the first few cycles and the incidence of reactions occurs mainly between the 7th and the 15th carboplatin infusion and may develop acutely during infusion or within minutes, hours, or days after the drug has been delivered (Genc et al., 2012).

Weekly dosing schedules of carboplatin have been identified as a risk factor for HSR in brain tumor patients (Yu et al., 2001). However, in the Canadian study (Lafay-Cousin et al., 2008), patients who received a weekly schedule had an incidence of HSR comparable to that of patients who were treated by monthly dosing, although HSR occurred 3 months earlier in the weekly dosing group.

According to Markman et al. (1999), the threshold for the manifestation of a reaction is expected to drop with each treatment because the patient is sensitized during the first treatment, and retreatment with the same drug provides the additional immunological stimulation necessary for a reaction.

Moreover, an increased severity of HSRs might be linked to re-exposure. Genc et al. (2012) reported seven out of eight patients had worsening hypersensitivity symptoms. Wiesner et al. (2004) observed grade III or IV reactions after re-exposure in five out of nine LGG patients with initial grade II reactions. The Canadian study found that the frequency of grade III and IV reactions rose from 18.2% at the first HSR episode to 41.7% for the second episode (Lafay-Cousin et al., 2008). Dodgshun et al. (2016) reported grade III reactions on rechallenge in two patients who initially experienced grade I and II reactions. In the study by Shah et al. (2016), 19 patients experienced a subsequent high-grade HSR (Grade III or IV) after initiating carboplatin rechallenge. In any case, Chang et al. (1995) did not document any increase in severity of HSR after re-exposure. The main issue is related to the possibility that an increased severity of hypersensitivity after re-exposure might expose the patient to the risk of anaphylaxis (Kook et al., 1998; Yu et al., 2001).

## Management

Chemotherapy protocols based on a combination of carboplatin and other drugs produced good results in terms of progression-free survival rate, as in LGG. Thus, when a patient shows a response to a carboplatin-based regimen, strategies that alter the HSRs may be justified as an attempt to salvage an effective therapy.

However, when HSRs occur early in treatment, it may be harder to predict the response to treatment and the benefit of continuing carboplatin may be inferior to the risk of a more severe allergic reaction. Therefore, according to International Society of Pediatric Oncology LGG protocol, in patients developing an allergy to carboplatin during consolidation, therapy shall be continued with alternative drug combinations (cisplatin/vincristine and cyclophosphamide/vincristine) (Azizi, 2010). The substitution of carboplatin with another platinum compound (such as cisplatin) may be limited by cross-reactivity of platinum-specific IgE (Pagani, 2010). The cisplatin and cyclophosphamide combination showed efficacy when alternated with carboplatin in the French “BABY-SFOP” LGG study (Rakotonjanahary et al., 2015). Despite its effectiveness, however, cisplatin is more ototoxic and nephrotoxic than carboplatin (Bertolini et al., 2004). In patients receiving carboplatin, the incidence of ototoxicity is approximately 7% (Dean et al., 2008; Jehanne et al., 2009). In patients receiving cumulative cisplatin doses of ≤200 mg/m^2^, 200–400 mg/m^2^, and ≥400 mg/m^2^, the incidence increases to 59, 68, and 65%, respectively (Peleva et al., 2014). Moreover, severe nephrotoxicity has not been reported during carboplatin-therapy and reduction of glomerular filtration rate to less than 50% is less frequent than with cisplatin. Moreover, cisplatin combined with cyclophosphamide could further damage renal function, therefore, in order to limit cumulative doses of these agents, no more than five cycles of both combinations shall be given. Therefore, to date the vincristine/carboplatin combination remains the most widely adopted multi-agent chemotherapy for childhood LGG. Of course, in patients sensitive to carboplatin the benefit of continuing this agent must be traded off against the risk of more severe reactions in view of the fact that no precise way to identify patients likely to react or to predict the severity of the reactions has yet been found.

To date, various desensitization and/or premedication protocols have been proposed, in children developing carboplatin HSR as a late event.

Pretreatment with steroids or antihistamines usually fails to prevent IgE-mediated reactions. The effectiveness of oral antihistamines has been found only in mild or moderate cases of carboplatin HSRs (Genc et al., 2012); moreover, the prolonged use of steroids is associated with long-term side effects in children and adolescents, such as mood changes, weight gain, and osteoporosis (Lafay-Cousin et al., 2008). The literature points to a low efficacy of premedication alone in re-exposure to carboplatin in patients with HSR. Aquino et al. (1999) and Heath et al. (2003) have reported complete failure of premedication (100% discontinuation rate). In Chang et al. (1995) and Wiesner et al. (2004) the effectiveness of premedication was 20 and 28.6%, respectively.

To date, several desensitization protocols for re-administering carboplatin have been implemented and have produced promising results (Markman et al., 1999; Lazzareschi et al., 2002; Lee et al., 2009; Castells et al., 2012) (**Table 1**). Drug desensitization is a procedure designed to obtain temporary clinical tolerance, especially in cases where the patient seems to have benefited from the drug (Castells, 2006; Lee et al., 2009; Castells et al., 2012). The complete target dose of the drug is administered in separate incremental steps in order to obtain an inhibition of mast-cell activation for the specific drug antigen. Sometimes this procedure is accompanied by a more intense premedication to prevent any risk of reaction (Genc et al., 2012). It is possible that the administration of small, increasing doses of antigen delivered at fixed time intervals may consume IgE antibodies slowly without acute reactions by inducing a prolonged hypo-responsiveness to triggering doses of the desensitizing antigen. Successful re-exposure to carboplatin is defined as the ability to complete the full planned course of treatment. All other cases where carboplatin is discontinued are considered a failure of re-exposure.

**Table 1:** Success rate of desensitization in patients with hypersensitivity reactions to carboplatin.

Studies	No. of patients treated with carboplatin	Patients with HSR (No./%)	Patients re-exposed to carboplatin (No./%)	No. of patients re-exposed with desensitization protocol	Success rate of desensitization (%)
Lazzareschi et al., 2002	29	6 (20.6%)	6 (100%)	6	6/6 (100%)
Lafay-Cousin et al., 2008	105	44 (41.9%)	34 (77.2%)	12	1/12 (8.3%)
Genc et al., 2012	50	20 (40%)	19 (95%)	9	6/9 (66.6%)
Dodgshun et al., 2016	59	16 (27.1%)	10 (62.5%)	10	2/10 (20%)
Shah et al., 2016	144	56 (38.9%)	55 (98.2%)	25	19/25(76%)

In Lazzareschi et al. (2002) described a successfully modified desensitization protocol for carboplatin administration in six children who had an allergic reaction to the drug. The protocol consisted of a standard dose of carboplatin (175 mg/m^2^ in 100 ml saline solution) at an increasing infusion rate, without premedication. The drug was administered every 30 min starting with a dose of 0.3 mg/m^2^/min and reaching 2.4 mg/m2/min in five steps (Lazzareschi et al., 2002) (**Table 2**). The protocol allowed the patients to receive carboplatin without adverse reactions in all re-treated patients. Genc et al. (2012) (**Table 2**) implemented a seven-step desensitization protocol in nine children affected by LGG, with a success rate of 66.6%. Patients were premedicated with pheniramine maleate (1 mg/kg/dose IV) and dexamethasone (0.3 mg/kg/dose IV) and the drugs were infused progressively every 15 min, beginning with a 0.1 mg/dose bolus, up to a 25 mg/dose. In the study by Shah et al. (2016) patients were re-exposed to therapy with carboplatin using precautionary measures, which included: prolongation of infusion (1–2 h); premedication with H1 antagonists; H2 antagonists, and corticosteroids. In patients with recurrent reactions despite precautionary measures, and in patients in which the first reaction was considered severe, a desensitization scheme was proposed which involved the administration of carboplatin in gradually increasing doses, from as low as 0.01 mg/min up to a maximum of 1.5 mg/min in nine progressive increments (**Table 2**). The success rate was 76%.

**Table 2:** Effective desensitization protocols.

Lazzareschi et al., 2002			Genc et al., 2012			Shah et al., 2016		
Step	Infusion rate (mg/m^2^/min)	Time (min)	Step	Infusion rate (mg/m^2^/min)	Time (min)	Step	Infusion rate (mg/m^2^/min)	Time (min)
1	0.3	30	1	0.1	15	1	0.01	15
2	0.6	30	2	1	15	2	0.1	15
3	1.2	30	3	2.5	15	3	0.5	15
4	1.8	30	4	5	15	4	1	15
5	2.4	30	5	10	15	5	1	15
			6	25	15	6	1	15
			7	Remaining	15	7	1	15
				dose		8	1.5	15
						9	1.5	15

However, according to Lafay-Cousin et al. (2008) and Dodgshun et al. (2016), the desensitization protocol demonstrated low efficacy, due to differences in starting dose, infusion rate and number of increments compared with other studies. In the Canadian study, the desensitization protocol consisted of a progressive increase in the rate of carboplatin infusion over various periods (from 1 h to 6 h) according to previously described schedules (doses of 1, 2.5, 5, 10, 25, and 50 mg of carboplatin infused at 1 mg/min every 15 min; the remainder of the dose was infused at the standard rate of 200 mg/h) (Broome et al., 1996; Ogle et al., 2002). Nevertheless, among the 12 patients who underwent desensitization and premedication (antihistamine with or without corticosteroid initiated from 3 days to 1 h before carboplatin infusion), only one patient (8.3%) was able to complete his carboplatin therapy (Lafay-Cousin et al., 2008). In this study the lower success rate of desensitization may be due to the starting dose being higher than in other studies (Lazzareschi et al., 2002; Genc et al., 2012; Shah et al., 2016). Dodgshun et al. (2016) reported a success rate of 20%. All patients received premedication with dexamethasone (between 0.05 and 0.15 mg/kg delivered via IV 1 h prior to carboplatin) and cetirazine (∼0.2 mg/kg delivered orally 1 h prior to carboplatin) and all but one, were exposed to the desensitization diagram with subsequent infusions of increasing doses of carboplatin according to the scheme described by Confino-Cohen et al. (2005). The protocol consisted of an initial infusion of 1/1000 of the total dose in the first 90 min, followed by 1/100 of the total dose for an additional 90 min, up to 1/10 in 90 min and the remainder of the dose in the last 90 min. Desensitization was not effective in this cohort. It is not clear why the success rate is so different from what was previously reported in adult literature. This may be due to the lack of a gradual increase in the rate of infusion (from 0.6 mg/m^2^/min to 5.5 mg/m^2^/min in the fourth step), and to the limited number of increments.

## Conclusion

As carboplatin has been used with increasing frequency for the management of childhood cancers, HSRs have emerged as a significant complication of the therapy although their features involving pediatric patients remain limited. HSRs occur mainly between the 7th and the 15th carboplatin infusion, therefore attention must be paid to the cumulative number of infusions rather than to the cumulative dose.

When a patient shows a response to a carboplatin-based regimen, strategies that alter the HSRs may be justified as an attempt to salvage an effective therapy, although the benefits of continuing the carboplatin chemotherapy should always be carefully weighed against the risks of potential dangerous complications.

Patients with an early onset allergic reaction should switch to alternative chemotherapy regimens that offer a good efficacy rate in order to avoid any risk associated with re-exposure. Thus, the likelihood of completing therapy is higher if restricted to patients with a late onset reaction.

Premedication with anti-histamines and/or corticosteroids is able to prevent an allergic reaction only in a limited number of patients with mild or moderate reactions. Several desensitization protocols have been implemented in order to re-administer carboplatin with various efficacy results. Hypothesis on their effectiveness to be further investigated are based on the potential role of the starting dose, infusion rate and number of increments. A lower starting dose, a slow infusion, and a number of increments greater than or equal to 4 are associated with a greater probability of success.

## Author Contributions

All authors listed, have made substantial, direct and intellectual contribution to the work, and approved it for publication.

## Conflict of Interest Statement

The authors declare that the research was conducted in the absence of any commercial or financial relationships that could be construed as a potential conflict of interest.
